# [Corrigendum] miR-382 inhibits migration and invasion by targeting ROR1 through regulating EMT in ovarian cancer

**DOI:** 10.3892/ijo.2024.5698

**Published:** 2024-09-25

**Authors:** Hong Tan, Qingnan He, Guanhui Gong, Yixuan Wang, Juanni Li, Junpu Wang, Ding Zhu, Xiaoying Wu

Int J Oncol 48: 181-190, 2016; DOI: 10.3892/ijo.2015.3241

Following the publication of the above article, an interested reader drew to the authors' attention that certain of the Transwell migration and invasion assay data panels shown in Figs. 3E and G and 7E and G on p. 1754 and 1757 respectively contained overlapping data panels, both within Fig. 3 and between Figs. 3 and 7, such that data which were intended to represent the results of differently performed experiments had apparently been derived from the same original sources. Specifically, the 'con' and 'pre-con' data panels in Fig. 3 were overlapping, as were the 'pre-con' and 'pcDNA.1-ROR1' panels comparing Fig. 3 with Fig. 7, and the Editorial Office subsequently pointed out to the authors that the 'con' and 'pre-con' data panels in Fig. 3E also contained an overlapping edge.

After having examined their original data, the authors realized that these figures were inadvertently assembled incorrectly. The corrected versions of Figs. 3 and 7 are shown on the next page, now showing the correct data for the 'con' experiment in Fig. 3E, the 'pre-con' experiment in Fig. 3G, and the 'pcDNA.1-ROR1' panel in Fig. 7G. 'The authors are grateful to the Editor of *International Journal of Oncology* for granting them the opportunity to publish this corrigendum, and all the authors agree with its publication; furthermore, they apologize to the readership of the journal for any inconvenience caused.

## Figures and Tables

**Figure 3:**
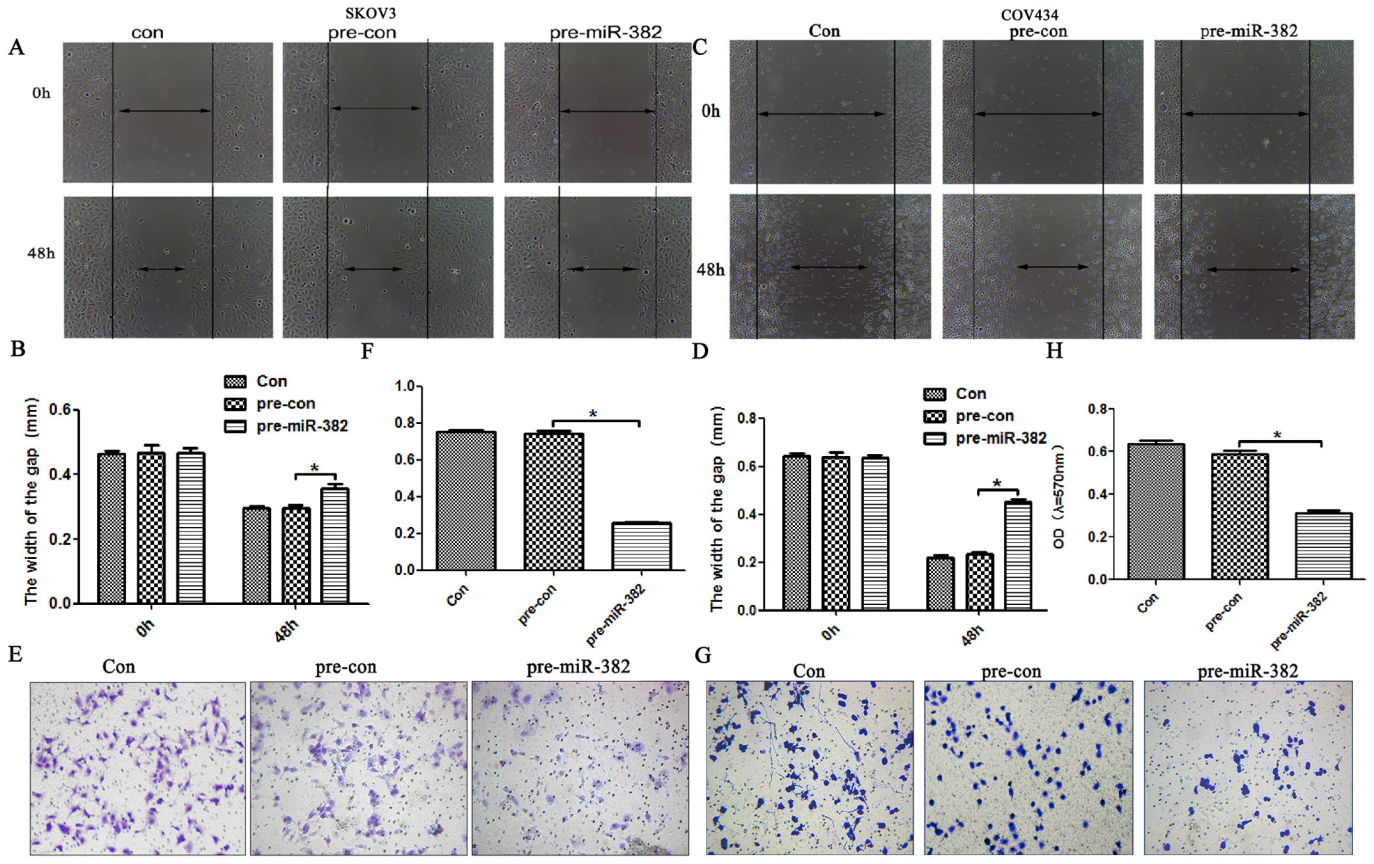
miR-382 inhibits the migration and invasion of ovarian cancer cells. (A and B) miR-382 inhibited the migration in SKOV3 cells as evaluated by wound scratch assay. (C and D) miR-382 suppressed the migration in COV434 cells. (E and F) miR-382 inhibited the invasion in SKOV3 cells in Transwell chamber invasion assays. (G and H) miR-382 restrained the invasion in COV434 cells in the Transwell assays. n=3, ^*^p<0.05.

**Figure 7:**
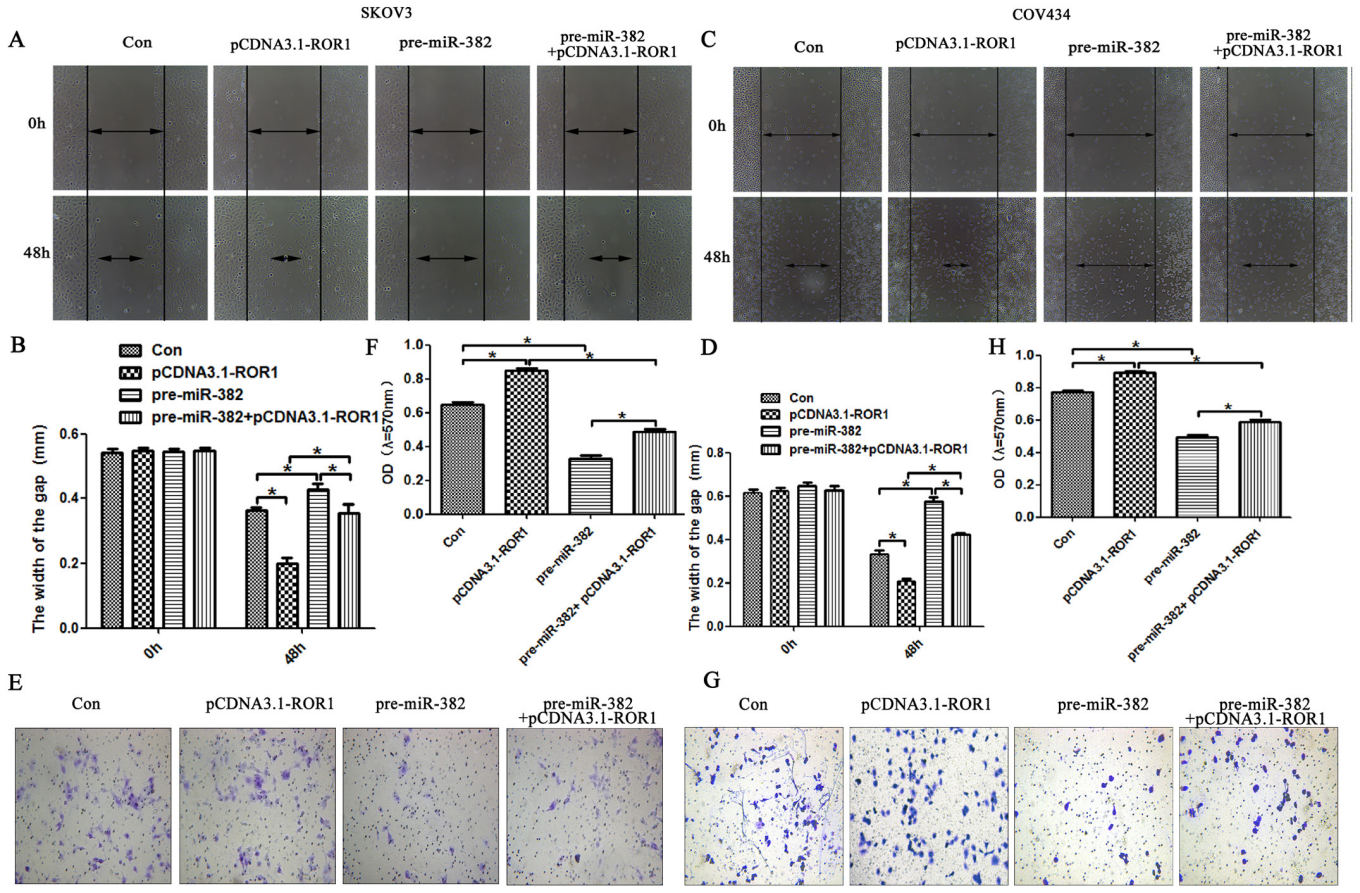
miR-382 rescued the promotion effect of ROR1 on migration and invasion of ovarian cancer cells. (A and B) ROR1 promoted migration by targeting miR-382 in SKOV3 cells as shown in the wound scratch assay. (C and D) ROR1 promoted migration by targeting miR-382 in COV434 cells in the wound scratch assays, (E and F) and promoted the invasion by targeting to miR-382 in SKOV3 cells. (G and H) miR-382 accelerated the invasion effect of ROR1 by targeting miR-382 in COV434 cells in the Transwell assays. n=3, ^*^p<0.05.

